# Frontostriatal circuitry as a target for fMRI-based neurofeedback interventions: A systematic review

**DOI:** 10.3389/fnhum.2022.933718

**Published:** 2022-08-24

**Authors:** Linda Orth, Johanna Meeh, Ruben C. Gur, Irene Neuner, Pegah Sarkheil

**Affiliations:** ^1^Department of Psychiatry, Psychotherapy and Psychosomatics, RWTH Aachen University, Aachen, Germany; ^2^Department of Psychiatry and Psychotherapy, University of Münster, Münster, Germany; ^3^Department of Psychiatry, Perelman School of Medicine, University of Pennsylvania, Philadelphia, PA, United States; ^4^Institute of Neuroscience and Medicine 4, Forschungszentrum Jülich, Jülich, Germany

**Keywords:** neurofeedback, real-time fMRI, connectivity neurofeedback, neuromodulation, frontostriatal circuitry

## Abstract

Dysregulated frontostriatal circuitries are viewed as a common target for the treatment of aberrant behaviors in various psychiatric and neurological disorders. Accordingly, experimental neurofeedback paradigms have been applied to modify the frontostriatal circuitry. The human frontostriatal circuitry is topographically and functionally organized into the “limbic,” the “associative,” and the “motor” subsystems underlying a variety of affective, cognitive, and motor functions. We conducted a systematic review of the literature regarding functional magnetic resonance imaging-based neurofeedback studies that targeted brain activations within the frontostriatal circuitry. Seventy-nine published studies were included in our survey. We assessed the efficacy of these studies in terms of imaging findings of neurofeedback intervention as well as behavioral and clinical outcomes. Furthermore, we evaluated whether the neurofeedback targets of the studies could be assigned to the identifiable frontostriatal subsystems. The majority of studies that targeted frontostriatal circuitry functions focused on the anterior cingulate cortex, the dorsolateral prefrontal cortex, and the supplementary motor area. Only a few studies (*n* = 14) targeted the connectivity of the frontostriatal regions. However, *post-hoc* analyses of connectivity changes were reported in more cases (*n* = 32). Neurofeedback has been frequently used to modify brain activations within the frontostriatal circuitry. Given the regulatory mechanisms within the closed loop of the frontostriatal circuitry, the connectivity-based neurofeedback paradigms should be primarily considered for modifications of this system. The anatomical and functional organization of the frontostriatal system needs to be considered in decisions pertaining to the neurofeedback targets.

## Introduction

Neurofeedback (NF) is a biofeedback method that enables individuals to modify the relevant neural targets for treatment purposes. Magnetic resonance imaging-based NF, which has been continuously advanced over the last two decades (Weiskopf, 2012; Sulzer et al., 2013; Watanabe et al., 2017), can induce an altered activation level in the targeted brain region or modify connectivity between different brain regions. The main goal of this effort is the amelioration of aberrant activation and connectivity patterns in clinical populations. The fundamental prerequisite for NF treatment is the selection of an appropriate target. NF techniques have been probed on various structures as a potential target for behavioral improvement and treatment of neuropsychiatric and movement disorders (Linden and Turner, 2016; Linhartová et al., 2019; Lipp and Cohen Kadosh, 2020; Anil et al., 2021). The search for an optimal NF target ought to involve the existing knowledge about the anatomical and functional organization of the underlying neurocircuitry. Various components of the frontostriatal circuitry (FSC) have been common targets for probing NF-induced modifications.

The FSC is known to be involved in a variety of affective, cognitive, and motor functions, underpinning complex human behavior (Bonelli et al., 2007; Beste et al., 2012). According to clinical studies, alterations within the FSC may underlie the pathophysiology of various psychiatric and neurological disorders, including major depressive disorder (MDD) (Baxter et al., 1989; Greicius et al., 2007), schizophrenia (Li et al., 2020), substance-use disorders (SUD) (Fettes et al., 2017), anxiety disorders including obsessive-compulsive disorder (OCD) (Graybiel and Rauch, 2000; Dunlop et al., 2016), post-traumatic stress disorder (PTSD), and eating disorders (Foerde et al., 2015). Additionally, abnormalities within this circuitry have been proposed to contribute to the pathophysiology of many primary movement disorders, such as Huntington's disease (HD), Parkinson's disease (PD), and tic disorders (Galvan et al., 2015; Blumenstock and Dudanova, 2020). It is therefore not surprising that the components of this circuitry, such as the prefrontal regions, the thalamus and the striatal nuclei, have been modification targets for trials probing new treatment strategies like transcranial magnetic stimulation (TMS) (Alkhasli et al., 2019; Lefaucheur et al., 2020) and deep brain stimulation (DBS) (Aum and Tierney, 2018; Andrade et al., 2020).

However, the question is which targets are effective in improving the symptoms being studied. Have prospective controlled trials already provided an answer to this question? Indeed, there is no universal consensus on the best target for NF in patients suffering from neuropsychiatric symptoms. On the other hand, NF interventions are highly dependent on precise localization of the target regions or networks (Linden et al., 2012). It is timely to make sure that the available knowledge about the organization of the FSC is well integrated into the selection of brain targets for NF modifications.

### Frontostriatal circuitry subsystems

**Figure 1:**
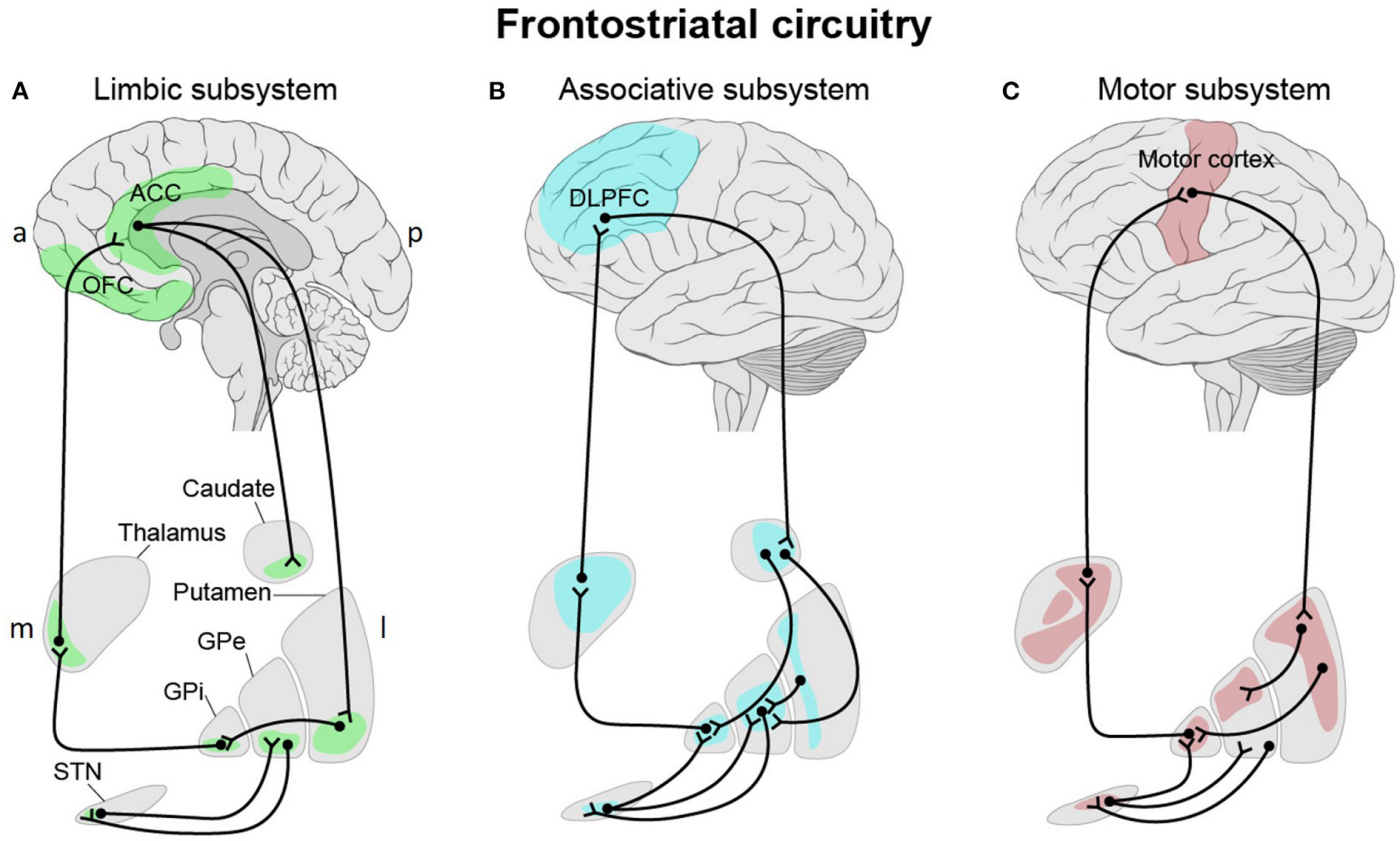
Three-part organization of the frontostriatal circuitry with distinct “limbic” **(A)**, “associative” **(B)**, and “motor” **(C)** subsystems linking the frontal cortical regions and the striatum. **(A)** The “limbic” subsystem is divided into two parts. The first part originates in the lateral orbitofrontal cortex (OFC) and projects to the ventromedial sector of the caudate nucleus. This region innervates the dorsomedial globus pallidus interna (GPi) and rostromedial substantia nigra (SNr, not shown). The latter projects to the ventral anterior thalamic nucleus, magnocellular part and the mediodorsal thalamic nucleus, magnocellular part before it forms a closed loop with the lateral OFC. Both the anterior cingulate cortex (ACC) and medial OFC project to the ventral striatum (ventromedial caudate, ventral putamen, nucleus accumbens and olfactory tubercle) which in turn project to the rostrolateral GPi and the rostrodorsal SNr. *Via* the mediodorsal thalamic nucleus, magnocellular part, the SNr sends fibers back to the ACC and medial OFC. **(B)** Within the “associative” subsystem, the dorsolateral prefrontal cortex (DLPFC) projections terminate in the dorsolateral head of the caudate nucleus. The caudate nucleus projects to the dorsomedial part of the GPi and globus pallidus externa (GPe) and from there to the rostral portions of the SNr. The GPi closes the loop *via* the parvocellular portion of the ventral anterior thalamic nucleus to the DLPFC. **(C)** Motor-relevant cortical areas (motor, premotor, supplementary motor, and somatosensory cortices) innervate the caudal putamen, which sends input to the ventrolateral GPi and GPe and to the caudolateral portions of the SNr. The GPi sends input to the ventrolateral nucleus of the thalamus, which in turns forms a closed loop with the motor cortex. In all three subsystems, the subthalamic nucleus (STN) modulates input to the thalamus *via* the GPi and/or GPe. a, anterior; p, posterior; m, medial; l, lateral. This figure adapted from Obeso et al. (2008).

The FSC forms a closed-loop system with direct projections from the frontal cortex to the striatum and indirect projections from the striatum (*via* the thalamus) back to the frontal cortex (Figure 1) (Öngür and Price, 2000). Already in 1986, Alexander et al. proposed a three-part organization, with distinct “limbic,” “associative,” and “motor” subsystems, within the circuitry linking the frontal cortical regions and the striatum (Alexander et al., 1986). Since then, subdivisions of the frontostriatal projection system have been extensively investigated based on various approaches through anatomical links, histochemical properties, patterns of gene expression and biochemical variations. The findings converge in a functional organization, subserving affective, cognitive, and motor functions (Basile et al., 2021). It is also likely to be topographically organized along medio-lateral and ventral-dorsal axes (Jarbo and Verstynen, 2015; Haber, 2016). The three major functional subsystems share common features and the main anatomical structures. All three circuits originate in the frontal cortex, connect with the striatum (putamen, ventral striatum, or caudate), project to the globus pallidus and the substantia nigra and from there form connections with the thalamus. Each subsystem forms a loop and connects back to the frontal cortex (Bonelli et al., 2007).

### Limbic subsystem

The anterior cingulate cortex (ACC) and the orbitofrontal cortex (OFC) (“limbic” subsystem) project most densely to the ventral striatum, which includes the ventromedial caudate, the ventral putamen, and the nucleus accumbens (Mega et al., 1997). The lateral OFC sends fibers to the ventromedial caudate nucleus (Tekin and Cummings, 2002; Barbas, 2007), while the medial OFC and the ACC project to the ventral striatum (ventromedial caudate, ventral putamen and nucleus accumbens) (Mega et al., 1997; Haber, 2003; Levy and Dubois, 2006; Bonelli et al., 2007).

The ACC, as part of the limbic and affective system, monitors cognitive regulation of emotions (Delevich et al., 2015). Additionally, this region seems to be involved in action selection and expression of emotion- and fear-related evaluation (Stevens et al., 2011). The OFC is a key brain area in emotional reappraisal and the generation of affective states (Fettes et al., 2017). Additionally, this brain area is involved in the representation of rewarded and non-rewarded values (Rolls, 2019), in reward-based learning (Kringelbach, 2005), and in reward prediction error (Boorman et al., 2013).

Neuroimaging studies have revealed abnormal activations in the ACC and the OFC leading to the dysregulation of their projections in the ventral striatum in various pathological conditions such as MDD (Biver et al., 1994; Frodl et al., 2010), OCD (Graybiel and Rauch, 2000; Menzies et al., 2008; Radua et al., 2010), substance-use disorders (Everitt and Robbins, 2005; Burton et al., 2015), schizophrenia (Wang et al., 2015), Tourette (Neuner et al., 2014; O'Neill et al., 2019), PTSD (Chen et al., 2019), and attention deficit hyperactivity disorder (ADHD) (Bledsoe et al., 2013).

### Associative subsystem

Neurons from the dorsolateral prefrontal cortex (DLPFC) send their input most densely to the dorsolateral head of the caudate nucleus (“associative” subsystem) (Parent et al., 1984; Parent and Hazrati, 1995).

The DLPFC plays a key node in dorsal attention networks, which supports basic cognitive selection of sensory information and responses (Corbetta and Shulman, 2002; Kuo and Nitsche, 2012). It is involved in executive functions including working memory, selective attention, control of cognitive processes (Curtis and D'Esposito, 2003), and decision making (Krawczyk, 2002). The “associative” subsystem is also involved in anticipation, monitoring, and use of feedback in task performance as part of executive function (Alvarez and Emory, 2006). Even though no direct projections to the emotion generating areas exist, the DLPFC influences emotional response (Ochsner et al., 2012).

Dysfunction within this circuitry has been associated with SUD (Abernathy et al., 2010; Hu et al., 2015), MDD (Koenigs and Grafman, 2009; Furman et al., 2011), schizophrenia (Callicott et al., 2000), OCD (Figee et al., 2013), eating disorders (Hayes et al., 2015), and PTSD (Ke et al., 2015).

### Motor subsystem

Neurons from the motor-related cortical areas (motor, premotor, supplementary motor, and somatosensory cortices) innervate the caudal putamen (“motor” subsystem) in a topographic pattern (Lehéricy et al., 2006; DeLong and Wichmann, 2007). This subsystem is mainly associated with planning, preparation, control, and execution of movement (Luppino and Rizzolatti, 2000; Nachev et al., 2008; Svoboda and Li, 2018).

Dysfunctions of this subsystem are associated with both common psychiatric disorders such as schizophrenia (Walther, 2015), MDD (Exner et al., 2009), OCD, and bipolar disorder (Hirjak et al., 2018), and major movement disorders such as PD (Galvan et al., 2015) and HD (Blumenstock and Dudanova, 2020). The abnormalities of this system seem to be an intersection between movement disorders and psychiatric conditions (Cummins et al., 2015). Frequently, affective disorders or psychosis predate the onset of motor symptoms in these patients (Ishihara and Brayne, 2006; Duff et al., 2007; Xu et al., 2016). Tourette's syndrome, a tic disorder with chronic motor and/or vocal tics and psychiatric impairments and comorbidities, also emphasizes this frontostriatal subsystem as an interface for neurological and psychiatric pathologies (Neuner et al., 2014).

The current review article is dedicated to NF interventions that aim at introducing changes in the activations of brain regions within the FSC. Several neuromodulation techniques have been investigated in recent decades, including invasive methods such as DBS (Dougherty, 2018) and non-invasive methods such as (repetitive) TMS (Lefaucheur et al., 2020), transcranial direct current stimulation (tDCS) (Palm et al., 2016), and electroconvulsive therapy (ECT) (Park et al., 2021). Almost all of these techniques have been focused on inducing activation changes in brain regions within the FSC. Various localizations have been tested to find an optimal stimulation target. However, multiple targets have been shown to be effective in improving the related symptoms (Sadleir et al., 2012; Honey et al., 2017; Barbour et al., 2019).

Unlike the direct neurostimulation techniques like DBS and TMS, functional magnetic resonance imaging (fMRI) NF enables targeting the connectivity of two or more brain regions, which may enhance the treatment effect (Watanabe et al., 2017). For this reason, fMRI NF, as a non-invasive neuromodulation technique, should be considered as a potential technique for neuromodulation of the FSC in treatment of psychiatric conditions and movement disorders. This technique is a form of biofeedback in which the participant receives real-time information about their ongoing brain activation, allowing for self-regulation training that can lead to clinical improvement and symptom reduction (Weiskopf, 2012). In a NF paradigm, participants learn to regulate their own neural activation guided by feedback to facilitate a desired neuropsychological pattern. Previous reviews have analyzed various mechanisms underlying NF (Sitaram et al., 2017; Shibata et al., 2019; Muñoz-Moldes and Cleeremans, 2020). Learning processes (Strehl, 2014) like operant conditioning (Birbaumer et al., 2013) and reinforcement learning (Lubianiker et al., 2022) have been broadly accepted as the neuropsychological mechanism for NF-based training. While learning control over specific neural substrates is assumed to underlie changing specific behaviors, the role of awareness (Stirner et al., 2022), metacognition and various forms of implicit and explicit learning in NF-based training have not been uncovered yet. Understanding the learning processes involved in NF in terms of frontostriatal functioning and monoaminergic modulation is essential for developing efficient NF interventions for brain and mental disorders. Recording of fMRI as an indirect measure of brain activity might be influenced by neurotransmitter modulation. Surprisingly, there is only one study that directly investigated the effect of NF on the endogenous release of dopamine (Ros et al., 2021). Imaging of the neurotransmission system in association with NF training using single-photon emission tomography and positron emission tomography might be leading the way to investigate the role of brain's key neuromodulatory systems in NF-based modulations.

In the early 2000s, electroencephalography (EEG) was the first and only method for providing real-time information about brain activation in the NF setting. However, the use of EEG does not permit accurate localization (Cohen et al., 2011). In particular, studying affective disorders using EEG may be inadequate given that abnormal brain activity is also found in subcortical areas such as the thalamus, the striatum, and the amygdala. Luckily, it has been shown that fMRI NF can be employed to detect changes in blood oxygen level-dependent (BOLD) activity in these brain regions of interest in real time (Sulzer et al., 2013). Due to its whole-brain coverage and high resolution, fMRI NF has gained considerable popularity over the past decade (Weiskopf, 2012), with 99 articles, according to a recent review (Thibault et al., 2018), having been published based on real-time fMRI (rt-fMRI) studies.

One of the main issues in designing NF intervention studies is the choice of target regions. The structural and functional correlates of most major psychiatric disorders are becoming better characterized, owing to expanding databases of neuroimaging studies and developing quantitative meta-analytic algorithms. Brain areas and networks within the FSC, which are affected across various psychiatric disorders (Casey et al., 1997; Cubillo et al., 2012; Pulcu and Elliott, 2015), may represent promising targets for NF intervention studies. Providing a systematic review of the studies that have investigated NF interventions based on target regions within the FSC, we aimed to address the following questions: (1) Which target regions within the FSC have been selected for rt-fMRI NF? (2) Which behavioral/clinical parameters have been addressed by the rt-fMRI NF targeting the FSC? Furthermore, issues pertaining to the design of rt-fMRI NF studies and suggestions for future studies are discussed.

## Methods

In this systematic review we followed the PRISMA (Preferred Reporting Items for Systematic Reviews and Meta-Analyses) guidelines (Moher et al., 2009).

### Information sources

We searched the following electronic databases for peer-reviewed studies published until February 22, 2022: Pubmed/MEDLINE, Web of Science/Web of Knowledge, EMBASE, Scopus, Cochrane Library, and PsycINFO. Further internet-based searches were carried out on the “Real-Time Functional Imaging and Neurofeedback database” and “ClinicalTrials.gov.” The Cochrane Library and ClinicalTrials.gov was used to identify currently ongoing or planned studies.

### Search strategy

The following search terms and syntax in title, abstract and keywords were used: (Neurofeedback OR Biofeedback) AND (fMRI OR “functional MRI” OR “functional magnetic resonance imaging”) AND (thalamus OR striat^*^ OR putamen OR accumbens OR cauda^*^ OR subthalamic OR pallidu^*^ OR “Olfactory tubercle” OR “substantia nigra” OR cingulate OR ACC OR OFC OR IFG OR ^*^PFC OR ^*^FRONTAL OR “^*^motor cortex” OR SMA). The search was conducted by the author LO.

### Eligibility criteria

All study designs applying fMRI-based NF techniques to modulate regions belonging to the FSC were considered. All pilot and feasibility studies, randomized controlled trials, clinical studies, and cohort and case control studies in original research format were selected (reviews, book chapters, and conference abstracts were excluded). We only included studies with humans with no limitations in sex, age, ethnicity, and nationality. Only studies published in the English language were included.

### Data selection

After removing duplicates, study selection was conducted by two reviewers (LO and JM). First, titles and/or abstracts of studies retrieved using the search strategy and those from additional sources were screened to identify studies that would potentially meet the inclusion criteria outlined above. Then full texts of these potentially eligible studies were retrieved and assessed for eligibility. Studies originating from the same author group and/or research group were carefully screened to avoid duplication of data. A total of 79 studies met the above-mentioned criteria and were considered for data analysis.

### Database

Data from the selected studies was extracted by the authors LO and JM and stored in an excel sheet based on the following template:

1. Information on study population

- sample size
- average age
- gender distribution
- healthy volunteers and/or clinical population
- (clinical) population specification

2. Information on the NF Intervention

- region(s) of interest (ROI)
- regulation direction (increase or decrease)
- number and duration of sessions and runs
- feedback timescale (intermittent or continuous), feedback type (numerical, social, scale, or curve)

3. Statistical differences

- between group comparison randomized
- between group comparison not randomized
- within group comparison randomized
- within group comparison not randomized

4. Control condition

- placebo control (yoked/computer-generated sham feedback, alternative ROI feedback)
- feedback from contralateral ROI
- feedback based on opposite regulation direction
- control without feedback intervention (with or without fMRI)
- no control condition
- other

5. Other study information

- blinding (single-blinded, double-blinded, or non-blinded; if a study did not specify blinding, it was also classified as not blinded)
- pre-registration

6. Imaging findings regarding the real-time NF [changes in brain activity and connectivity of the target region(s)]
7. *Post-hoc* imaging findings pre and post NF intervention: Whole-brain changes in brain activity and connectivity
8. Behavioral outcome of the NF intervention
9. Clinical outcome of the NF intervention (if any)
10. Follow-up effects of NF intervention (if any)

All extracted data were mutually checked by LO and JM. Conflicting results were discussed among authors to achieve a consensus.

Tables 1–**4** show the aforementioned extracted data. Additional information on the studies is provided in Supplementary Table 1.

**Table 1:** Details of real-time fMRI neurofeedback studies with regulation target in the “limbic subsystem.”

**References**	**ROI(s), regulation direction and definition**	**Study population**	**Control condition**	**Feedback**	**Regulation of target ROI(s)/online changes**	**Offline analysis (whole brain)**	**(*Post-hoc*) connectivity changes**	**Behavioral/clinical changes**
Cordes et al., 2015	ACC (bilateral) ↑; %	11 schizophrenia patients (11 healthy control volunteers)	Other (HV)	Continuous (social)	Yes, ACC↑	STG, pre-/postcentral gyri, l. MTG, l. IPG↑	r. SMG, r. MTG	Not reported
Dyck et al., 2016	ACC (bilateral) ↑; %	3 schizophrenia patients (no controls)	No control	Continuous (scale)	Yes, ACC ↑	Reward system ↑	-	Clinical improvement
Li et al., 2013	ACC (bilateral) and MPFC (right) ↑(MPFC)↓(ACC); #	10 nicotine-dependent smokers (no controls)	No control	Continuous (scale)	~Yes, ACC↓; No, MPFC →	Occipital, middle cingulate, parietal cortex ↑ (During upregulation blocks)	-	Behavioral improvement
Zilverstand et al., 2017	ACC (dorsal) ↑; #	7 ADHD patients (6 controls)	Control without feedback intervention (with fMRI)	Continuous (scale)	Yes, dACC↑ (both groups)	-	-	Clinical improvement
Mathiak et al., 2015	ACC (dorsal) ↑; %	12 healthy volunteers (12 controls)	Other	Continuous (social)	Yes, dACC↑ (EG>CG)^*1^	Lateral occipital, striatum, DLPFC↑; insula, post-central gyrus, PCC↓ (EG/CG); putamen, IFG, l. occipital gyrus, l. MTG↑ (EG)	-	No behavioral improvement
Harmelech et al., 2013	ACC (left dorsal) ↑; #	20 healthy volunteers (no controls)	No control	Continuous (auditory)	Yes, l. dACC↑	IPL, SFG, MFG, MTG ↑	l. dACC → SFG, cingulate, LTC, IFG, IPS, PCC	No observation
deCharms et al., 2005	ACC (rostral) ↑↓; #	8 chronic pain patients and 8 healthy volunteers (4 control patients and 28 healthy volunteers)	Other	Continuous (curve)	Yes, rACC ↑↓ (EG)	ACC, SMC, insula, SMA, superior cerebellum, STG ↑ (EG)	-	Clinical improvement
Guan et al., 2015	ACC (rostral) ↑↓; #	8 post-herpetic neuralgia patients (6 control patients)	Placebo control	Continuous (scale)	Yes rACC ↑↓ (EG)	-	-	Clinical improvement
Rance et al., 2014a	ACC (rostral); pIns (left) ↑ (increase difference); #	10 healthy volunteers (no controls)	No control	Continuous (scale)	~ Yes, Insula↑↓; No, ACC →	IFG, l. thalamus, caudate↑	-	No behavioral improvement
Rance et al., 2014b	ACC (rostral); pIns (left) ↑↓; #	10 healthy volunteers (no controls)	No control	Continuous (scale)	Yes, Insula↑↓; ACC~	-	-	No behavioral improvement
Weiskopf et al., 2003	ACC (rostral-ventral and dorsal) ↑; %	1 healthy volunteer (no controls)	No control	Continuous (curve)	Yes, ACC ↑	ACC, SMA, basal ganglia↑	-	Behavioral improvement
Hamilton et al., 2011	ACC (subgenual) ↓; #	8 female healthy volunteers (9 controls)	Placebo control	Continuous (curve)	Yes sgACC ↓ (EG)	-	sgACC → l. PCC/cuneus (EG)	No observation
Klöbl et al., 2020	ACC (subgenual) ↓; #	6 healthy volunteers (6 controls)	Other	Continuous (social)	Yes, sgACC↓	Cerebellum, SMA, anterior insula, temporal lobes, anterior thalamus, putamen, caudate nucleus↑ SMC, FP, DMN, hippocampus, posterior thalamus, pons↓	-	Behavioral improvement
Hanlon et al., 2013	ACC (ventral) and DMPFC ↓(ACC)↑(DMPFC); #	15 nicotine-dependent smokers (no controls)	No control	Intermittent (scale)	Yes, vACC↓, No, DMPFC →	-	-	Behavioral improvement
Mathiak et al., 2010	ACC ↑; %	1 healthy volunteer (no controls)	No control	Continuous (social)	Yes, ACC↑	-	-	No observation
Zweerings et al., 2018	ACC ↑; %	9 PTSD patients (9 controls)	Other (HV)	Continuous (social)	Yes, ACC↑ (EG and HV)	l. IFG, STG, l. IPL↑	-	Clinical improvement
MacDuffie et al., 2018	ACC ↑↓; #	13 MDD patients (no controls)	Other	Intermittent (scale)	-^*2^	-	-	No observation
Canterberry et al., 2013	ACC ↓; #	9 nicotine-dependent smokers (no controls)	No control	Continuous (scale)	Yes, ACC↓	-	-	Clinical improvement
Tinaz et al., 2018	Connectivity between DMFC and insula (right) ↑; #	8 Parkinson's disease patients (no controls)	No control	Intermittent (scale)	Yes, DMPFC → insula↑	-	DMPFC → insula	No clinical improvement
Koush et al., 2017	Connectivity between DMPFC and bilateral amygdala ↑; #	9 healthy volunteers (6 controls)	Placebo control	Intermittent (numerical)	Yes, DMPFC → amygdala (EG)	DMPFC↑, r. amygdala↓ (EG), l. amygdala↑ (EG)	DMPFC → amygdala	Behavioral improvement
Zahn et al., 2019	Connectivity between SATL (right) and sgACC (anterior) ↑; #	14 MDD patients (14 controls)	Other	Continuous (scale)	Yes, r. SATL → sgACC↑ (EG)	-	r. SATL → sgACC (EG)	Clinical improvement
Jaeckle et al., 2021	Connectivity between SATL (right) and sgACC (anterior) ↓; %	19 MDD patients (16 controls)	Control without feedback intervention (without fMRI)	Continuous (scale)	Yes, r. SATL → sgACC↓ (EG)	-	r. SATL → sgACC	Clinical improvement (both groups)
Garrison et al., 2021	MPFC (bilateral) ↑; %	21 healthy volunteers (18 controls)	Placebo control	Continuous (scale)	Yes, MPFC ↑	-	MPFC → DLPFC/paracingulate cortex, Within fronto-parietal network, thalamus, caudate, LP and occipital cortex	Behavioral improvement
Li et al., 2018	NAcc (bilateral) ↑; %	19 female healthy volunteers (5 controls)	Placebo control	Continuous (scale)	Yes, NAcc↑	-	NAcc → VMPFC, reward circuit (EG)	Behavioral improvement (EG)
Greer et al., 2014	NAcc (bilateral) ↑↓; %	25 healthy volunteers (no controls)	Feedback based on opposite regulation direction/ Control without feedback intervention (with fMRI)	Continuous (scale)	Yes, NAcc ↑	-	NAcc → MPFC	Behavioral improvement
Scheinost et al., 2013	OFC (bilateral) ↑↓; #	10 anxiety patients (10 controls)	Placebo control	Continuous (curve)	-	-	Limbic area, prefrontal areas	Clinical improvement
Kirsch et al., 2016	Ventral striatum ↓; #	13 heavy drinking students [25 controls (2 control groups)]	Placebo control/control without feedback intervention (with fMRI)	Continuous (scale)	Yes, ventral striatum↓ (EG)	Prefrontal regions↑ (EG/CG) r. IFG↑ (EG)	-	No behavioral improvement
Mayeli et al., 2020	VMPFC (bilateral) ↑; %	18 healthy volunteers (9 controls)	Placebo control	Continuous (scale)	No, VMPFC →	MPFC, MTG, IFG, precuneus↓	-	No observation

The publications are sorted based on the region(s) of interest.

ADHD, attention deficit hyperactivity disorder; CG, control group; EG, experimental group; fMRI, functional magnetic resonance imaging; HV, healthy volunteers; MDD, major depressive disorder; NF, neurofeedback; PTSD, post-traumatic stress disorder; ROI, region of interest; ACC, anterior cingulate cortex; DLPFC, dorsolateral prefrontal cortex; DMFC, dorsomedial frontal cortex; DMPFC, dorsomedial prefrontal cortex; DMN, default mode network; FP, frontal parietal; IFG, inferior frontal gyrus; IPG, inferior parietal gyrus; IPL, inferior parietal lobe; IPS, intraparietal sulcus; LP, lateral parietal cortex; LTC, lateral temporal cortex; MFG, middle frontal gyrus; MPFC, medial prefrontal cortex; MTG, middle temporal gyrus; NAcc, nucleus accumbens; OFC, orbitofrontal cortex; pIns, posterior insula; PCC, posterior cingulate cortex; SATL, superior anterior temporal lobe; SFG, superior frontal gyrus; SMA, supplementary motor area; SMC, somatomotor cortex; SMG, supramarginal gyrus; STG, superior temporal gyrus; VMPFC, ventromedial prefrontal cortex; d, dorsal; r, rostral; sg, subgenual; v, ventral; l., left; r., right.

# functional localizer; %, anatomical ROI; ↑, up-regulation; ↓, down-regulation; ↑↓, bidirectional regulation; (ROI1) → (ROI2), connectivity between two regions; -, no information; ~, mixed results.

*1 Social reward led to stronger activity in the ACC compared to standard feedback.

*2 Regulation was possible, but not the main interest of the study.

For the sake of completeness, we also surveyed pre-registered studies, which have yet to publish results. The corresponding information is presented in Supplementary Table 2.

## Results

**Figure 2:**
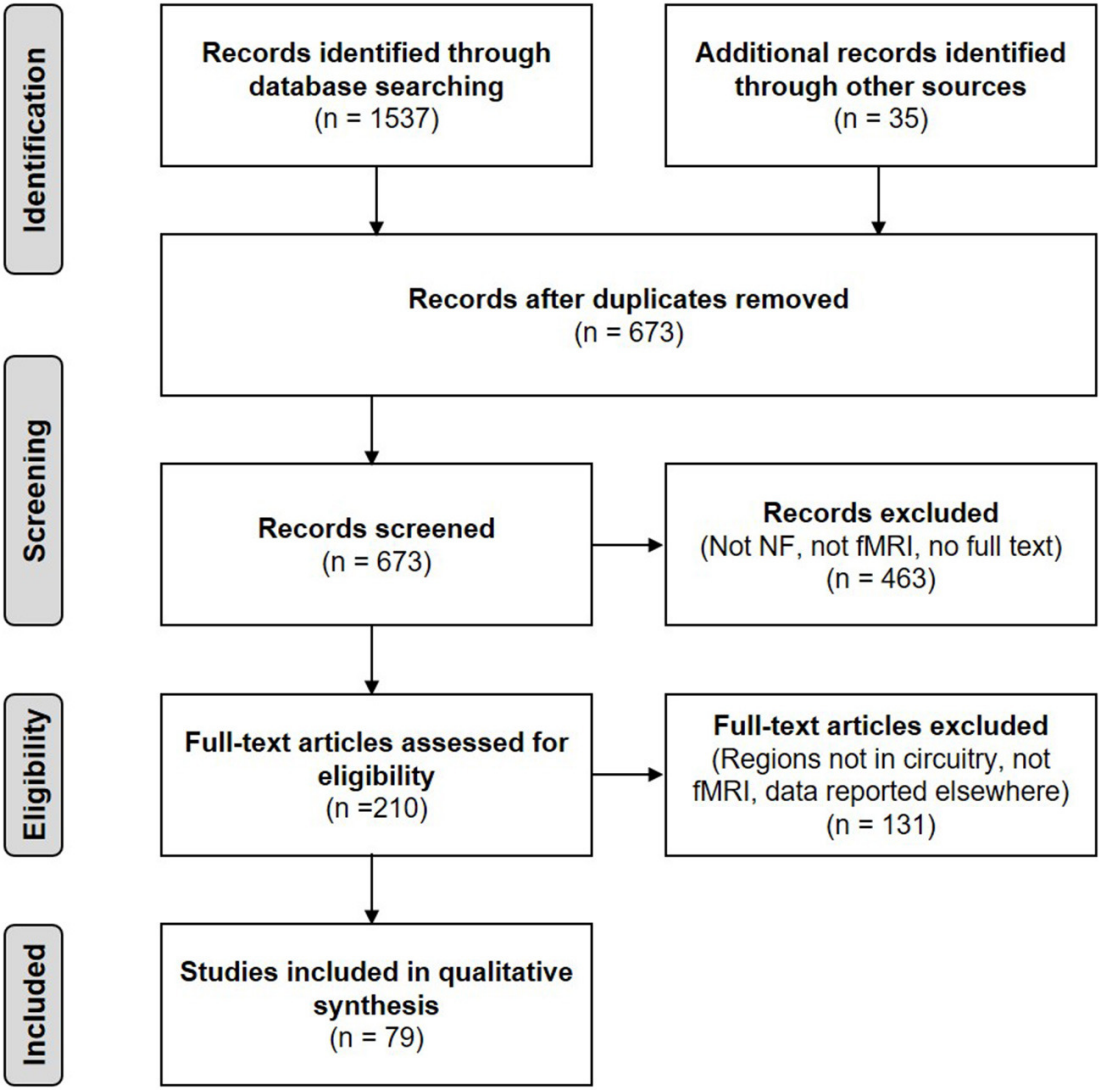
PRISMA flow diagram of the literature search (adapted from Moher et al., 2009).

Our initial search retrieved 1,537 citations. After eliminating duplicates, 673 articles were assessed based on the search criteria in their title and/or abstract. Thirty-five additional records could be identified through other sources such as reference lists. A total of 463 articles were excluded because they did not meet the inclusion criteria. The final number of studies included in the review was 79. Figure 2 shows a flow diagram of the article selection process.

The majority of studies originate from the last 10 years (2012–2021) (*n* = 68, 86%), with the first publication investigating fMRI-based NF in the SMA in 2003 (Weiskopf et al., 2003). Based on our search criteria, the survey confirmed a growing tendency, which has been growing steadily over the past decade, toward research in rt-fMRI NF (Tursic et al., 2020).

### Study populations

**Figure 3:**
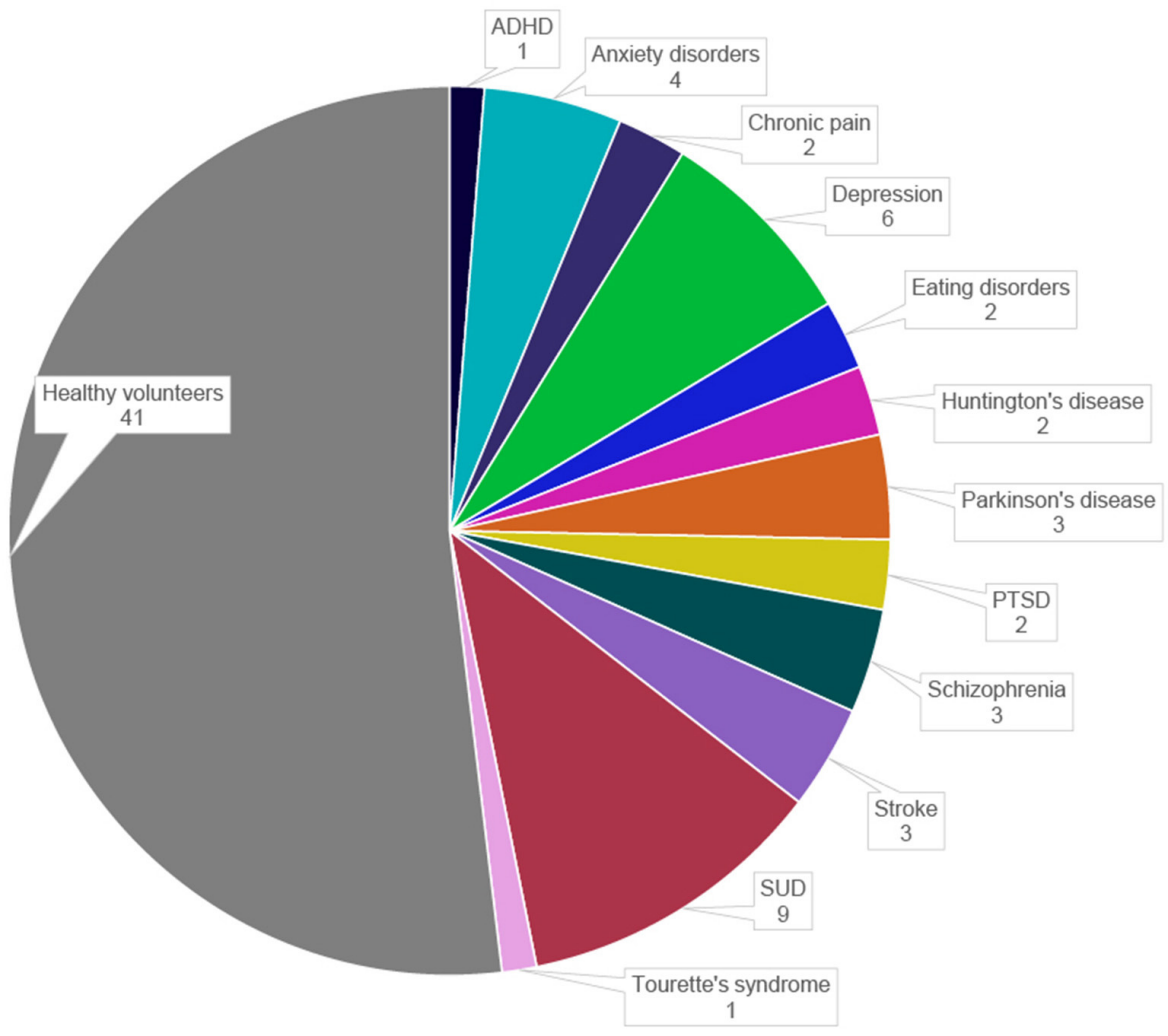
Distribution of study populations in the selected publications. ADHD, attention deficit hyperactivity disorder; PTSD, post-traumatic stress disorder; SUD, substance-use disorders.

The data on study populations were extracted from the selected publications into major categories. In most publications, the potential of rt-fMRI NF is investigated in healthy volunteers (*n* = 41, 52%). The clinical populations include SUD (*n* = 9, 11%), depression (*n* = 6, 8%), anxiety disorders (*n* = 4, 5%), schizophrenia (*n* = 3, 4%), PD (*n* = 3, 4%), paralysis after stroke (*n* = 3, 4%), PTSD (*n* = 2, 3%), eating disorders (*n* = 2, 3%), HD (*n* = 2, 3%), chronic pain disorders (*n* = 2, 3%), ADHD (*n* = 1, 1%), and Tourette's syndrome (*n* = 1, 1%) (Figure 3). The total number of participants ranges from 1 to 76 with the sample sizes of the published studies growing over the last 10 years.

### Age of the participants

In the clinical groups the age of the participants was various with the mean age of 38 years for depression, about 20 years for anxiety, 30 years for eating disorders, 37 years for schizophrenia, 37 years for ADHD, and 43 years for PTSD. Participants in NF studies with neurological diseases were older and aged above 39 years for PD, above 49 years for stroke and around 50 years for HD. In the NF studies that examined patients with chronic pain, the average age was around 50 years. In the study investigating NF in patients with Tourette's syndrome the average age was 16. In one of the studies with anxiety patients, age was not mentioned (Scheinost et al., 2013).

### Targets for NF intervention

**Figure 4:**
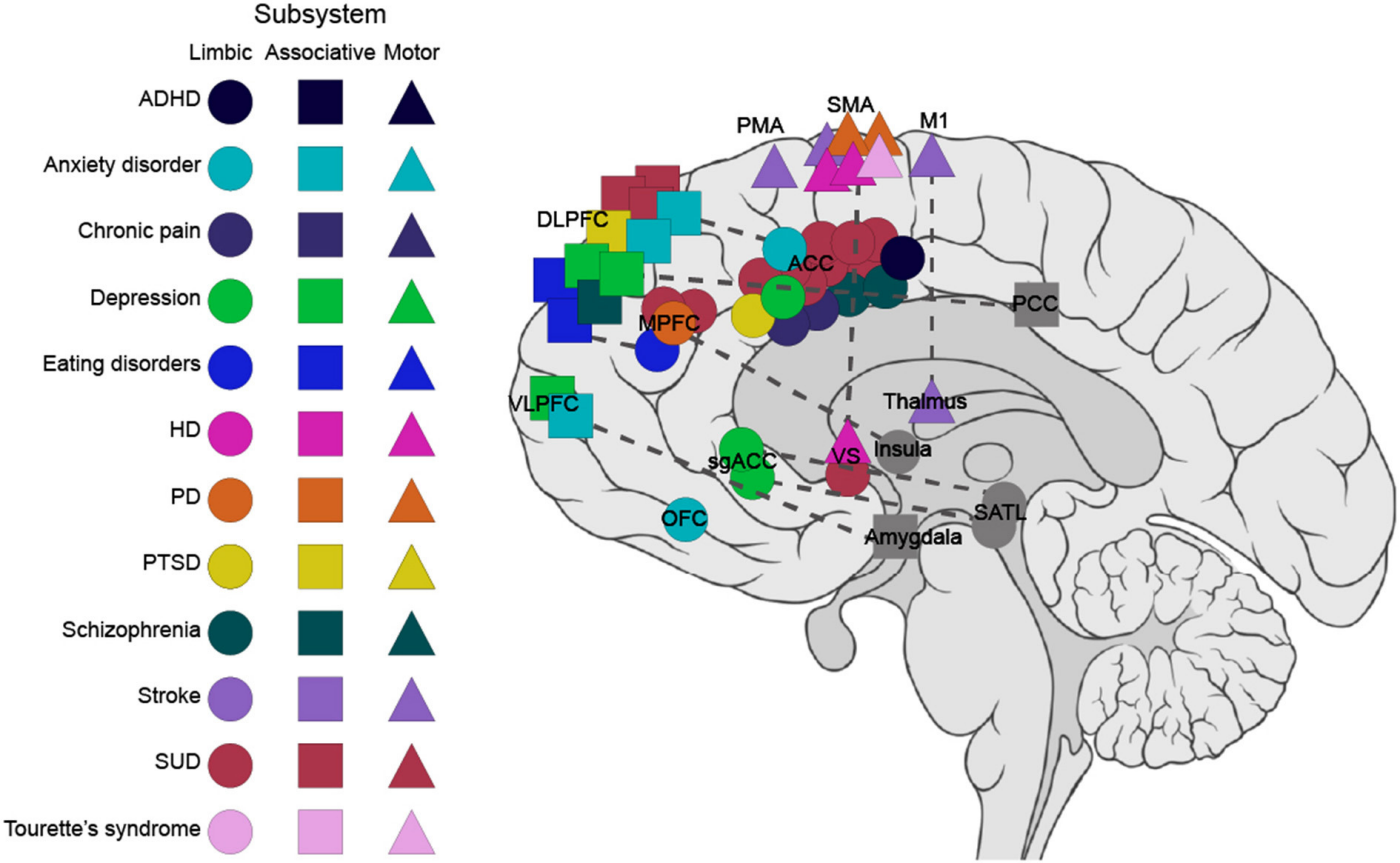
Targets within the frontostriatal circuitry (FSC) for fMRI-based NF in clinical populations. This graph depicts the targeted regions within the FSC for the three subsystems. The circle, square, and triangle refer to the regions in limbic, associative, and motor subsystems, respectively. The dashed line represents connectivity feedback. Regions outside the FSC with connectivity feedback to FSC regions are marked in gray. ACC, anterior cingulate cortex; ADHD, attention deficits hyperactivity disorder; DLPFC, dorsolateral prefrontal cortex; HD, Huntington's disease; IFG, inferior frontal gyrus; M1, primary motor cortex; MPFC, medial prefrontal cortex; OFC, orbitofrontal cortex; PCC, posterior cingulate cortex; PD, Parkinson's disease; PMA, premotor area; PTSD, post-traumatic stress disorder; SATL, superior anterior temporal lobe; sgACC, subgenual cingulate; SMA, supplementary motor area; SUD, substance-use disorders; VS, ventral striatum; VLPFC, ventrolateral prefrontal cortex.

Target regions were selected from all three subsystems of the FSC (Figure 4). The most studied target region in the publications is the ACC (*n* = 22, 28%) belonging to the limbic subsystem (Table 1), followed by the supplementary motor area (SMA) (*n* = 8, 10%) belonging to the motor subsystem (Table 3). The associative subsystem (Table 2) has been investigated in 16 publications (27%), targeting different parts of the prefrontal cortex (PFC), the DLPFC and the ventrolateral PFC, and the inferior frontal gyrus (IFG). Most of the studies have focused on the frontal side of the FSC with only four publications (5%) investigating NF modulation in subcortical components of the FSC, namely the striatum (Greer et al., 2014; Kirsch et al., 2016; Li et al., 2018) and the thalamus (Zotev et al., 2018). Notably, 18% of the publications have investigated the effect of NF on interconnections of the FSC regions (*n* = 14).

**Table 2:** Details of real-time fMRI neurofeedback studies with regulation target in the “associative subsystem.”

**References**	**ROI(s), regulation direction and definition**	**Study population**	**Control condition**	**Feedback**	**Regulation of target ROI(s)/online changes**	**Offline analysis (whole brain)**	**(*Post-hoc*) connectivity changes**	**Behavioral/clinical changes**
Lisk et al., 2020	Connectivity between DLPFC (left) and amygdala (left) ↑; #	27 female healthy volunteers (no controls)	No control	Continuous (scale)	~	-	-	No behavioral improvement
Taylor et al., 2022	Connectivity between DLPFC (left) and PCC (left) ↑; #	19 volunteers with subclinical levels of depression (no controls)	Placebo control	Intermittent (scale)	Yes, l. DLPFC → l. PCC↑	-	DLPFC → PCC	Clinical improvement
Weiss et al., 2022	Connectivity between DLPFC and striatum ↑; %	20 healthy volunteers (20 controls)	Placebo control	Continuous (scale)	No, DLPFC → striatum →	-	-	No observation
Zhao et al., 2019	Connectivity between VLPFC (right) and amygdala (right) ↑; #	23 male anxiety patients (no controls)	Placebo control	Continuous (scale)	Yes, VLPFC → amygdala↑ (EG)	-	VLPFC → amygdala	Clinical improvement (EG)
Kohl et al., 2019	DLPFC (left) ↑; #	16 overweight or obese participants (19 controls)	Placebo control	Continuous (scale)	Yes, l. DLPFC↑ (EG); VC↑ (CG)	l. DLPFC ↑ (CG)	DLPFC → VMPFC	Behavioral improvement (both groups)
Sherwood et al., 2016	DLPFC (left) ↑; #	18 healthy volunteers (7 controls)	Control without feedback intervention (without fMRI)	Continuous (curve)	Yes, l. DLPFC↑ (EG)	-	-	Behavioral improvement
Takamura et al., 2020	DLPFC (left) ↑; #	6 MDD patients (no controls)	No control	Continuous (curve)	~	-	-	Clinical improvement
Zhang et al., 2013	DLPFC (left) ↑; #	15 healthy volunteers (15 controls)	Placebo control	Continuous (scale)	Yes, l. DLPFC↑ (EG)	DLPFC, PPC, l. middle occipital gyrus↑ (EG)	-	Behavioral improvement
Travassos et al., 2020	DLPFC (left) ↑↓; #	17 healthy volunteers (no controls)	No control	Continuous (scale)	Yes, DLPFC↑↓	Insula, cingulate cortex, PMA, thalamus, dorsal striatum↑ (upregulation)	-	No observation
Van den Boom et al., 2018	DLPFC (left) ↑↓; #	13 healthy volunteers (11 controls)	Placebo control	Continuous (visual)	Yes, DLPFC↑↓ (EG)	-	-	No observation
Zilverstand et al., 2015	DLPFC (left) ↑ and insula (right) ↓; #	9 female anxiety patients (9 controls)	Control without feedback intervention (with fMRI)	Intermittent (scale)	Yes, DLPFC↑ (EG/CG), insula↓ (EG)	-	-	Clinical improvement (EG)
Zweerings et al., 2019	IFG (left) and pSTG (left) ↑↓; #	21 schizophrenia patients and 35 healthy volunteers	Feedback based on opposite regulation direction	Intermittent (numerical)	Yes, l. IFG and l. pSTG ↑↓	-	l. IFG/ l. pSTG → IPL, PCC/precuneus, MPFC (Pat.)	No clinical improvement
Rota et al., 2009	IFG (right) ↑; #	7 healthy volunteers (5 controls)	Placebo control	Continuous (scale)	Yes, r. IFG↑ (EG)	l. rolandic operculum, putamen, insula, l. medial FC, l. STG, ACC, SFG, SMA, cerebellum ↑	-	Behavioral improvement (EG)
Sarkheil et al., 2015	LPFC (left) ↑; #	8 healthy volunteers (6 controls)	Control without feedback intervention (with fMRI)	Intermittent (numerical)	No, l. LFPC →	Amygdala↓ (EG)	l. LPFC → r. PFC, PCC (EG); l. LPFC → r. amygdala	No behavioral improvement
Zweerings et al., 2020	PFC (left) ↑; #	20 PTSD patients (21 controls)	Control without feedback intervention (with fMRI)	Intermittent (numerical)	No, l. PFC ↓	Amygdala ↑, left IFG↓	-	Clinical improvement (PTSD)
Keller et al., 2021	VLPFC (left or right crossover) ↑; %	39 MDD patients and 37 healthy volunteers	Feedback from contralateral ROI	Intermittent (numerical)	Yes, VLPFC↑ (l. VLPFC>r. VLPFC)	PFC, precentral gyrus, SMA, MCC, occipital areas, SPL, thalamus, cerebellum↑ (MDD and HV); Cingulate↑(MDD)	-	Clinical improvement

The publications are sorted based on the region(s) of interest.

CG, control group; EG, experimental group; fMRI, functional magnetic resonance imaging; HV, healthy volunteers; MDD, major depressive disorder; NF, neurofeedback; PTSD, post-traumatic stress disorder; ROI: region of interest; ACC, anterior cingulate cortex; DLPFC, dorsolateral prefrontal cortex; IFG, inferior frontal gyrus; IPL, inferior parietal lobe; LP, lateral parietal cortex; LPFC, lateral prefrontal cortex; MCC, middle cingulate gyrus; MPFC, medial prefrontal cortex; MTG, middle temporal gyrus; PCC, posterior cingulate cortex; PFC, prefrontal cortex; PMA, premotor area; SFG, superior frontal gyrus; SMA, supplementary motor area; SPL, superior parietal lobe; STG, superior temporal gyrus; VC, visual cortex; VLPFC, ventrolateral prefrontal cortex; VMPFC, ventromedial prefrontal cortex; d, dorsal; p, posterior; r, rostral; s, subgenual; v, ventral; l., left; r., right.

# functional localizer; %, anatomical ROI; ↑, up-regulation; ↓, down-regulation; ↑↓, bidirectional regulation; (ROI1) → (ROI2), connectivity between two regions; -, no information; ~, mixed results.

**Table 3:** Details of real-time fMRI neurofeedback studies with regulation target in the “motor subsystem.”

**References**	**ROI(s), regulation direction and definition**	**Study population**	**Control condition**	**Feedback**	**Regulation of target ROI(s)/online changes**	**Offline analysis (whole brain)**	**(*Post-hoc*) connectivity changes**	**Behavioral/clinical changes**
Megumi et al., 2015	Connectivity between M1 (left) and LP (left) ↑; %	12 healthy volunteers (21 controls (2 control groups))	Placebo control/control without feedback intervention (with fMRI)	Intermittent (numerical)	Yes, l. M1 → l. LP↑ (EG)	-	DMN (LP, PCC, MPFC) → MVN (M1, SMA, IPS, FEF) (EG)	No observation
Yamashita et al., 2017	Connectivity between M1 (left) and LP (left) ↑; %	18 healthy volunteers (12 controls)	Feedback based on opposite regulation direction	Intermittent (scale)	Yes, M1 → LP↑ (EG), M1 → LP↓(CG)	-	M1 → LP	~
Liew et al., 2016	Connectivity between M1 and ipsilesional thalamus ↑; #	4 chronic stroke patients (no controls)	No control	Continuous (scale)	Yes, M1 → thalamus (3/4)	-	Perilesional M1 → ipsilesional thalamus	No observation
Chiew et al., 2012	M1 (bilateral) ↑↓ (increase laterality); #	13 healthy volunteers (5 controls)	Placebo control	Continuous (scale)	~	Basal ganglia, thalamus, cortical motor regions, parietal cortex, premotor, SMA, r. AI, ACC ↑ (NFvs.Rest)	-	No observation
Berman et al., 2012	M1 (left) ↑; #	15 healthy volunteers (no controls)	No control	Continuous (scale)	No	Thalamus, SPL, IPS, anterior insula, IFG, MFG ↑	-	No observation
Blefari et al., 2015	M1 (left) ↑; #	11 healthy volunteers (no controls)	No control	Continuous (scale)	~	SMA, PMA, putamen, caudate, IPL ↑; MTG, MFG, precuneus, insula, MOG ↓	-	No behavioral improvement
Yoo et al., 2008	M1 (left) ↑; #	11 healthy volunteers (11 controls)	Placebo control	Continuous (curve)	Yes, M1↑ (EG)	Pre-/post-central gyrus, r. parahippocampal gyrus, MTG↑ (EG), Follow-up: hippocampus and the limbo-thalamo-cortical pathway↑ (EG)	-	No observation
Yang et al., 2021	M1 (left) or PMA (left ventral) ↑; %	15 + 15 healthy volunteers (no controls) ^*1^	Other	Continuous (scale)	~ No, l. M1 → (EG1); Yes, l. PMA↑ (EG2)	r. precentral cortex, l. SMA, l. rolandic operculum, r. IPL↑	-	No observation
Mehler et al., 2019	M1 and SMA (bilateral) ↑; #	17 healthy volunteers (no controls)	Other	Continuous (scale)	~ Yes, SMA↑, No, M1↓	-	-	No observation
Pereira et al., 2019	PMA (bilateral) interhemispheric connectivity ↑↓; #	10 healthy volunteers (no controls)	Control without feedback intervention (with fMRI)	Continuous (scale)	Yes, PMA↑↓	PMA, SMA, IFG, lentiform nucleus, cerebellum↑	l. PMA → r. PMA	No observation
Sitaram et al., 2012	PMA (left ventral) ↑; #	2 chronic stroke patients (4 healthy control volunteers)	Other (HV)	Continuous (scale)	Yes, PMA↑	PMA, SMA, SMC, IFG, medial FC, occipital gyrus↑	-	No clinical improvement
Marins et al., 2015	PMA (left) ↑; %	14 healthy volunteers (14 controls)	Placebo control	Continuous (scale)	Yes, l. PMA↑ (EG)	PMA, SFG, MFG, hippocampus, SMA, basal ganglia, cerebellum ↑ (EG)	-	No observation
Xie et al., 2015	PMA (right dorsal) ↑; #	12 healthy volunteers (12 controls)	Placebo control	Continuous (curve)	-	-	l. PMA → r. PPL, r. PMA → r. PPL↑	No observation
Hui et al., 2014	PMA (right) ↑; #	12 healthy volunteers (12 controls)	Placebo control	Continuous (curve)	Yes, PMA↑ (EG)	SMA, l. M1/S1, PPL, cerebellum↑	r. PMA → l. PPL	Behavioral improvement
Kober et al., 2019	Precentral gyrus (left lateral) ↑; #	11 healthy volunteers (no controls)	No control	Continuous (scale)	Yes, l. precentral gyrus↑	Cerebellum, pre-/post-central regions, SMA, basal ganglia, visual brain regions ↑	-	No observation
Mehler et al., 2020	SMA (bilateral) ↑; #	4 MCA stroke patients (no controls)	No control	Continuous (scale)	~	-	-	No observation
Papoutsi et al., 2018	SMA (bilateral) ↑; #	10 Huntington's disease patients (no controls)	No control	Continuous (scale)	Yes, SMA↑	l. putamen ↑	SMA → l. putamen, SMA → cerebellum	Clinical improvement
Sepulveda et al., 2016	SMA (bilateral) ↑; #	10 male healthy volunteers (10 controls)	Other	Continuous (scale)	Yes, SMA↑	Precentral gyrus, insula, supramarginal gyrus↑	MFG → SFG, l. ACC → r. ACC, l. SMA → r. SMA, l. precentral gyrus → r. precentral gyrus, l. SMA → precentral gyrus	No observation
Scharnowski et al., 2015	SMA (bilateral) and PHC (left) (Difference between the ROI) ↑↓; #	7 healthy volunteers (no controls)	Feedback based on opposite regulation direction	Continuous (scale)	Yes, SMA/PHC↑↓	SMA, PHC, Middle cingulate, l. SPL, r. SFG, precuneus↑	No changes	Behavioral improvement
Al-Wasity et al., 2021	SMA ↑; #	10 healthy volunteers (10 controls)	Placebo control	Continuous (scale)	Yes, SMA ↑ (EG); SMA ↓ (CG)	SMA, PMA, IPL, basal ganglia ↑ (EG); PMA, basal ganglia, middle frontal gyrus, r. IPL↑ (CG)	-	Behavioral improvement
Subramanian et al., 2011	SMA ↑; #	5 Parkinson's disease patients (5 controls)	Placebo control	Continuous (scale)	Yes, SMA↑ (EG)	SMA, PCG, STN, thalamus, GPi, insula, cerebellar vermis↑	-	Clinical improvement (EG)
Subramanian et al., 2016	SMA ↑; #	13 Parkinson's disease patients (13 controls)	Control without feedback intervention (without fMRI)	Continuous (scale)	Yes, SMA↑ (EG)	Cerebellum, frontal areas, putamen, insula, subthalamic nucleus, ACC↑	-	Clinical improvement (EG)
Hampson et al., 2011	SMA ↑↓; #	8 healthy volunteers (no controls)	No control	Continuous (curve)	Yes, SMA↑↓	-	SMA → striatum, thalamus	No observation
Sukhodolsky et al., 2020	SMA ↑↓; #	21 Tourette's syndrome patients (no controls)	Placebo control	Continuous (curve)	No, SMA →	r. putamen, caudate, dorsal frontal cortex↑ (during Upregulation, EG)	-	Clinical improvement (EG)
Papoutsi et al., 2020	SMA or SMA and striatum (left) ↑; #	16 Huntington's disease patients (16 controls)^*2^	Placebo control	Continuous (activity-based) Intermittent (FC-based) (scale)	Yes, SMA ↑ (EG1); SMA → striatum↑ (EG2)	-	SMA → striatum (EG2)	No clinical improvement
Auer et al., 2015	SMC (left and right) ↑; #	16 healthy volunteers (16 controls)	Control without feedback intervention (without fMRI)	Continuous (scale)	Yes, SMC ↑	SMC ↑ (EG)	-	No observation
deCharms et al., 2004	SMC (left) ↑; #	6 healthy volunteers (3 controls)	Placebo control	Continuous (curve)	Yes, SMC ↑ (EG)	Cerebellum, occipital, frontal regions ↑	-	No observation

The publications are sorted based on the region(s) of interest.

CG, control group; EG, experimental group; fMRI, functional magnetic resonance imaging; HV, healthy volunteers; MCA, middle cerebral artery; NF, neurofeedback; ROI, region of interest; ACC, anterior cingulate cortex; AI, anterior insula; DMN, default mode network; FEF, frontal eye fields; IFG, inferior frontal gyrus; IPL, inferior parietal lobe; IPS, intraparietal sulcus; LP, lateral parietal cortex; M1, primary motor cortex; MFG, middle frontal gyrus; MOG, middle occipital gyrus; MPFC, medial prefrontal cortex; MTG, middle temporal gyrus; MVN, medial visual network; PCC, posterior cingulate cortex; PHC, parahippocampal cortex; PMA, premotor area; PPL, posterior parietal lobe; S1, primary sensory area; SFG, superior frontal gyrus; SMA, supplementary motor area; SMC, somatomotor cortex; SPL, superior parietal lobe; d, dorsal; r, rostral; s, subgenual; v, ventral; l., left; r., right.

# functional localizer; %, anatomical ROI; ↑, up-regulation; ↓, down-regulation; ↑↓, bidirectional regulation; (ROI1) → (ROI2), connectivity between two regions; -, no information; ~, mixed results.

*1 This study compared NF from two motor regions.

*2 This study had two experimental and two control groups.

### Laterality

Most studies have used bilateral ROIs (*n* = 23, 29%), mainly targeting medial regions such as the ACC, the SMA, and the medial PFC (MPFC). Regions in the left hemisphere (*n* = 23, 29%) have been used significantly more often than in the right hemisphere (*n* = 6, 8%). Regions targeted in the left hemisphere mainly include the DLPFC and the motor regions such as the primary motor cortex (M1) and the premotor area (PMA). Eight studies (10%) have targeted multiple regions located in different hemispheres or have bilateral and lateral ROI. Only 36 of the 61 studies mentioned have clearly indicated the choice of laterality. In 18 studies (23%), participants received NF from an individually localized region. Laterality is either not mentioned or is different among participants in these studies.

### Experimental design

Control conditions are considered to be essential for demonstrating specific NF effects in clinical trials (Sorger et al., 2019). The majority (*n* = 58, 73%) of the selected studies apply a method for controlling the unspecific effects of NF interventions (Tables 1–4). No control group or control condition is chosen in 27% of the studies (*n* = 21) with only 18% of the publications being pre-registered (*n* = 14). For more details and additional information on the study population, see Supplementary Table 1.

**Table 4:** Details of real-time fMRI neurofeedback studies with regulation target in mixed subsystems.

**References**	**ROI(s), regulation direction and definition**	**Study population**	**Control condition**	**Feedback**	**Regulation of target ROI(s)/online changes**	**Offline analysis (Whole brain)**	**(*Post-hoc*) connectivity changes**	**Behavioral/ clinical changes**
Hartwell et al., 2016	ACC or PFC ↓; #	21 nicotine-dependent smokers (23 controls)	Control without feedback intervention (with fMRI)	Continuous (scale)	Yes, ACC or PFC↓ (EG)	-	-	Behavioral improvement
Karch et al., 2015	ACC, DLPFC or insula (DLPFC for HV) ↓; #	13 patients with AUD and 14 healthy volunteers (2 control patients and 5 control volunteers)	Placebo control and other (HV)	Continuous (scale)	Yes, targeted regions↓ (AUD, EG)	ACC, DLPFC, insula, ITG, medial FC, cuneus, parietal cortex↓ (AUD, EG)	l. ACC → thalamus, l. insula → MPFC, SFG, parietal ares, r. insula → OFC, medial FC, temporal ares, l. MFG → DLPFC, lentiform nucleus, thalamus, r. MFG → insula (AUD, EG)	Behavioral improvement (trend)
Karch et al., 2019	ACC, DLPFC or insula ↓; #	22 nicotine-dependent smokers (14 controls^*1^)	Placebo control	Continuous (scale)	Not reported	No NF-specific effect reported	-	No behavioral improvement
Karch et al., 2021	ACC, DLPFC or insula ↓; #	24 alcohol-dependent patients (24 controls)	Placebo control	Continuous (scale)	Yes, targeted regions↓ (EG)	ACC, medial FC, pre-/post-central gyrus, insula, caudate↓ (EG), cuneus, precuneus, inferior/medial occipital gyrus↑ (EG)	-	Clinical improvement (both groups)
Morgenroth et al., 2020	Connectivity between ACC (bilateral) and DLPFC (left) ↑; #	15 anxiety patients (15 controls)	Placebo control	Continuous (scale)	Yes, ACC → DLPFC↑ (EG)	-	DLPFC → SMA (EG); ACC, insula, inferior PFC, angular gyrus, SFG, PCC	Clinical improvement
Spetter et al., 2017	Connectivity between DLPFC and VMPFC ↑; #	8 obesity patients (no controls)	No control	Continuous (scale)	Yes, DLPFC → VMPFC↑	Insula, IFG, DLPFC, striatum↑	DLPFC → VMPFC	No behavioral improvement
Kim et al., 2015	ROI1: ACC/MPFC/OFC; ROI2: PCC/precuneus ↑; %	7 nicotine-dependent smokers (7 controls)	Other	Continuous (visual)	Yes, ROI1 → ROI2↑(FC-Group); ROI1↑ (Activity-Group)	-	ROI1 → precuneus, PCC, mOFC, ACC (FC Group); ROI2 → precuneus, PCC, mOFC, ACC, mOFC (FC Group)	No behavioral improvement
Zotev et al., 2018	Thalamus (anterior nucleus and the mediodorsal nucleus) ↑; %	15 healthy volunteers (14 controls)	Placebo control	Continuous (scale)	Yes, AN/MD ↑ (EG)	-	MD → precuneus, IFG, ACC, precentral gyrus, SN (EG). AN → caudate, lentiform nucleus (EG)	No observation

The publications are sorted based on the region(s) of interest.

AUD, alcohol use disorder; CG, control group; EG, experimental group; fMRI, functional magnetic resonance imaging; HV, healthy volunteers; NF, neurofeedback; ROI, region of interest; ACC, anterior cingulate cortex; AN, anterior nucleus; DLPFC, dorsolateral prefrontal cortex; FC, frontal cortex; IFG, inferior frontal gyrus; ITG, inferior temporal gyrus; MD, mediodorsal nucleus; MFG, middle frontal gyrus; mOFC, medial orbitofrontal cortex; MPFC, medial prefrontal cortex; OFC, orbitofrontal cortex; PCC, posterior cingulate cortex; PFC, prefrontal cortex; SFG, superior frontal gyrus; SMA, supplementary motor area; SN, substantia nigra; VMPFC, ventromedial prefrontal cortex; d, dorsal; r, rostral; s, subgenual; v, ventral; l., left; r., right.

# functional localizer; %, anatomical ROI; ↑, up-regulation; ↓, down-regulation; ↑↓, bidirectional regulation; (ROI1) → (ROI2), connectivity between two regions; -, no information; ~, mixed results.

* 1In this article data of control group was not evaluated. Data of experimental group was split into relapsed (n = 12) and non-relapsed group (n = 10).

Among the controlled studies (*n* = 58), blinding as concealment of group allocations and a method to overcome performance biases (Pildal et al., 2007) has been implemented in 31 studies. Twenty-three studies report being single-blinded with 8 incorporating double-blinding. Twenty-one studies do not include a control condition, precluding any possible indication with respect to blinding. Thirty-two of the 58 controlled studies were randomized.

Barring one study (Harmelech et al., 2013) with auditory feedback, all studies provided a visual feedback to shape the regional activation in the desired direction. While most studies implemented continuous feedback (*n* = 67, 85%), intermittent feedback (every 12–153 s) was also used frequently (Tables 1–4). Commonly, the feedback was some form of scale like a thermometer (*n* = 53, 67 %), although other feedback forms such as social feedback (emotional faces), numeric values, and graphs demonstrating the feedback course (as a curve) were also employed.

## Discussion

As a form of neuromodulation, fMRI NF has been investigated as a new therapy method in a number of proof-of-principle studies and clinical trials involving individuals with disorders such as schizophrenia, depression, anxiety disorders, and SUDs. This is a welcome development in treatment of mental health disorders given the limited effectiveness of current treatment recommendations, i.e., psychotherapy and pharmacotherapy (Leichsenring et al., 2022), and the high number of difficult-to-treat cases. That being said, the target brain regions for NF applications are still unclear. Fortunately, there is decades of functional neuroimaging research that can underpin NF target selection. The frontostriatal circuit that underlies a variety of affective, cognitive, and motor functions (Haber, 2016) has been identified as a NF training target for improving psychiatric deficits. Based on our systematic review, we provide an overview of the choice of behavioral/clinical targets, ROI and study design, discuss the findings and shortcomings, and make suggestions for future research.

### Targets within the FSC for FMRI-based NF

The treatment of different symptoms may require different NF targets with different underlying brain circuits (Figure 4). Identifying the appropriate targets is a central issue with respect to treatment efficacy. Previous NF studies have targeted various FSC structures distributed in all three subsystems. The ACC, the SMA and the DLPFC are the most commonly chosen regions as NF targets in the FSC (Tables 1–4). The selection of these structures has been supported mainly by neuroimaging findings of their anatomical and functional positions.

#### Anterior cingulate cortex

The ACC is the most studied region in the publications reviewed here. Extensive connections to the medial frontal cortex, the posterior cingulate cortex (PCC), the anterior medial temporal lobe, the dorsal medial thalamus, the nucleus accumbens and the brainstem nuclei (Rolls, 2019) indicate the ACC as a central node in the large-scale neural networks that may be dysfunctional in patients, either directly or through a downstream structure (Monosov et al., 2020).

In almost all the reviewed studies, which targeted the ACC for NF interventions, regulations of the targeted ROIs were achieved. The efficacy of NF training in the ACC to ameliorate behavioral and clinical symptoms, i.e., craving (Canterberry et al., 2013; Hanlon et al., 2013; Li et al., 2013; Karch et al., 2015; Hartwell et al., 2016), hallucination severity and affective state (Dyck et al., 2016), and pain perception (deCharms et al., 2005; Guan et al., 2015) was also demonstrated (Tables 2, 4).

NF interventions in the ACC also induced changes in the activity pattern of other regions, which were revealed in the *post-hoc* whole-brain analyses. Activity changes in relation to ACC modulations were observed in the thalamus (Rance et al., 2014a; Klöbl et al., 2020), the striatum (Rance et al., 2014a; Mathiak et al., 2015; Dyck et al., 2016), the PCC (Mathiak et al., 2015), and the insula (deCharms et al., 2005; Klöbl et al., 2020), suggesting a broad interaction with large-scale brain networks such as the default mode network (DMN) and the salience network (SN) (Uddin, 2015).

On the whole, the ACC within the limbic subsystem of FSC plays a significant role in mediating cognitive influences on emotion. As noted above, over- or under-activation of regions within the ACC and its connection appears to be associated with particular psychopathologies. Furthermore, NF modulations of the ACC were possible and were effective in reducing some of the aberrant behaviors. This evidence converges on the conclusion that the ACC should be considered as an effective NF target, enhancing the cognitive control improvements particularly in dysregulated emotional states.

#### Supplementary motor area

The SMA is a cortical region in the dorsomedial frontal cortex and just anterior to the primary motor cortex that contributes to movement (Kaas and Stepniewska, 2002). With connections to the limbic system, the ACC, the basal ganglia, the cerebellum, the thalamus, and the superior parietal lobe (Nguyen et al., 2014; Bozkurt et al., 2017), the SMA complex is thought to play a role in the initiation and coordination of movements (Nachev et al., 2008).

An altered SMA activity has been measured in movement disorders like HD (Klöppel et al., 2009), PD (Nachev et al., 2008), and Tourette's syndrome (Neuner et al., 2014), a psychiatric condition with tic disorders. Movement disturbance has also been regarded as an essential feature of depression (Sobin and Sackeim, 1997) and has been described in other psychiatric disorders like schizophrenia (Walther and Strik, 2012). Aberrant SMA activity profiles have been shown in depression (Sarkheil et al., 2020).

Understandably, the efficacy of SMA modulations through NF was investigated in several of the reviewed studies (Subramanian et al., 2011, 2016; Papoutsi et al., 2018, 2020; Mehler et al., 2019, 2020). Following NF training, SMA activity was found to be successfully regulated by the study populations afflicted with PD (Subramanian et al., 2011, 2016) and HD (Papoutsi et al., 2018, 2020) with improvements in motor performance. The clinical symptoms of Tourette's syndrome also showed improvements after NF training of SMA activity (Sukhodolsky et al., 2020).

Some *post-hoc* whole-brain analyses tapped into the SMA-striatal connections within the FSC. Several studies that targeted the SMA with NF paradigms showed activity changes in the striatal regions (Hampson et al., 2011; Scharnowski et al., 2015; Subramanian et al., 2016; Papoutsi et al., 2018; Sukhodolsky et al., 2020; Al-Wasity et al., 2021). An attenuated functional connectivity of the SMA-striatal neurocircuitry has already been reported in depression (Sarkheil et al., 2020). Altogether, these findings highlight a potential for NF modulations of the motor subsystem within the FSC to improve the (psycho)motor symptoms of psychiatric disorders.

#### Dorsolateral prefrontal cortex

The importance of the DLPFC within the FSC has been outlined in the reviewed studies. Besides its role in attention, cognitive control, and executive function, this region is involved in emotional response and has extensive connections to the thalamus, the dorsal caudate nucleus, the hippocampus, the OFC, and the posterior temporal, parietal, and occipital areas (Kobayashi, 2009).

The controlling role of this area has motivated its selection as a target region for NF modulations in the context of SUD (Karch et al., 2015), MDD (Takamura et al., 2020), anxiety disorders (Zilverstand et al., 2015), eating behavior in obesity (Spetter et al., 2017; Kohl et al., 2019), and in improving working memory in healthy participants (Sherwood et al., 2016).

The corresponding studies have demonstrated successful regulations of the DLPFC. Further *post-hoc* analyses have revealed changes on the whole brain level, e.g., the dorsal striatum, the thalamus, the parietal cortex, the occipital cortex, and the cuneus (Zhang et al., 2013; Kohl et al., 2019; Travassos et al., 2020; Karch et al., 2021).

The improvement of clinical and behavioral parameters was reported in almost all studies (Zhang et al., 2013; Karch et al., 2015; Sherwood et al., 2016; Kohl et al., 2019; Morgenroth et al., 2020; Takamura et al., 2020; Taylor et al., 2022). As for the long-term effects, considerable responsiveness and clinical improvement were observed 4 weeks (Kohl et al., 2019) and 3 months (Zilverstand et al., 2015; Karch et al., 2021) after NF training, which suggest a promising potential for NF in treatment of clinical conditions.

#### Subcortical regions

The subcortical components of the FSC and their connections have been extensively described based on the available functional and anatomical knowledge (Smith et al., 2014). NF studies have already contributed to the identification of the frontostriatal connections by showing alterations in the thalamostriatal regions linked to the NF modulations of the frontal cortical areas (Hampson et al., 2011; Rance et al., 2014a; Klöbl et al., 2020; Travassos et al., 2020; Garrison et al., 2021).

In comparison to NF training of the frontal cortical regions, few researchers have investigated whether humans can voluntarily control the striatal activity. This is probably due to the technical challenges like small size, dynamic activity changes, and deep subcortical location. The ventral striatum, as a key part of the reward system, is particularly interesting for feedback-based paradigms as it is related to learning by means of reward feedback (O'Doherty, 2004) and predicting rewards (Knutson and Cooper, 2005).

Given the importance of the ventral striatum in psychiatric disorders such as schizophrenia (Sorg et al., 2013), depression (Pan et al., 2017), and ADHD (Plichta and Scheres, 2014), three studies investigated the NF training of the ventral striatum. Two studies investigated the feasibility of NF training in healthy participants (Greer et al., 2014; Li et al., 2018), demonstrating successful voluntary regulation of the ventral striatum and resultant improvement in motivation and positive arousal. *Post-hoc* connectivity analyses revealed connectivity changes within the reward circuit (ventromedial frontal cortex and MPFC) (Knutson and Greer, 2008). Kirsch et al. used the ventral striatum as a regulation target to reduce craving in non-addicted heavy drinking students (Kirsch et al., 2016) and showed that participants were able to successfully regulate activation of the ventral striatum, with a transfer effect in measurement runs without feedback.

The thalamus, as part of the FSC, was probed as a target for NF training during retrieval of happy autobiographical memories (Zotev et al., 2018). The anterior and mediodorsal nuclei of the thalamus were chosen based on their involvement in episodic memory function and activation during recall of autobiographical memories. This group also investigated the potential of thalamus NF training and its connection to posterior alpha EEG power. Participants were able to significantly increase the BOLD activity of the thalamus nuclei, which were correlated with increased EEG alpha power indicating their involvement in the DMN (Sestieri et al., 2011). Overcoming the technical challenges, the aforementioned studies demonstrate the potential for NF modulations in subcortical regions within the FSC. Given the importance of subcortical regions within the FSC in many psychiatric disorders, more NF research in these areas should follow.

#### Frontostriatal connectivity as NF target

Connectivity feedback has been used in the context of anxiety, depression, PD, HD, and paralysis after stroke. Only 18% of studies have applied this method. Studying connectivity is closely linked to functional imaging methods like fMRI. The intersection between brain connectivity and fMRI-based NF is growing and has been recognized in the NF literature (Ruiz et al., 2014). With functional imaging, it is technically possible to consider the remote impact on brain regions connected to the NF target. For example, the connectivity within the frontostriatal system, including the MPFC-ventral striatum circuitry, has been shown to be facilitated by ventral striatum NF training (Greer et al., 2014; Li et al., 2018). The SMA-striatal connectivity has been shown to be influenced by SMA NF (Hampson et al., 2011).

Connectivity imaging allows the targeting of two or more regions, instead of focusing in the local activity of isolated brain regions, to achieve improvement. Two of the reviewed studies investigated the modulation of connectivity between the subgenual ACC and the superior anterior temporal lobe in MDD patients (Zahn et al., 2019; Jaeckle et al., 2021), demonstrating successful connectivity modulation and clinical improvements. In their recent study, Morgenroth et al. (2020) used a connectomic approach for ACC-based NF to investigate the potential of modulating the connectivity between the ACC and the DLPFC in patients suffering from high levels of trait anxiety. The patients were able to increase this connectivity and thereby improve clinical symptoms.

Connectivity NF, as the concept of using neuromodulation to target distributed brain networks is in line with the previous findings regarding different treatments strategies in normalizing pathological functional connectivity, including pharmacotherapy (Goveas et al., 2011; Abbott et al., 2013; Gudayol-Ferré et al., 2015), repetitive TMS (Beynel et al., 2020), ECT (Perrin et al., 2012), and DBS (Figee et al., 2013).

#### Laterality

Cerebral lateralization refers to the functional specialization of the two cerebral hemispheres (Geschwind and Galaburda, 1985). The left cerebral cortex is dominant for motor control and verbal processing, whereas the right cerebral cortex is dominant for spatial cognition, visualization, and depth perception (Mutha et al., 2012). The left and right hemispheres also have different functions in relation to emotions (Silberman and Weingartner, 1986). While the left hemisphere is responsible for handling positive emotions, the right hemisphere is more responsible for controlling emotional expressions, recognizing emotions, and negative emotions (Sackeim et al., 1982). A laterality of the thalamostriatal brain regions can also be assumed given the critical role of information integration and processing in the cortical motor and cognitive functions. There is considerable neurobiological (Glick et al., 1982; Cheesman et al., 2005), structural (Kooistra and Heilman, 1988) and electrophysiological (Eitan et al., 2013) evidence to suggest a laterality of the thalamostriatal brain regions.

Accordingly, it is not only the choice of the ROI but also the laterality that is critical for the NF effects. Our review indicates that researchers have investigated mainly left-sided ROIs and the lateralized effects of NF have not been adequately studied. Some studies have used a functional localizer to determine the target region without reference to a rationale of the choice of laterality.

### Effectivity of NF modulations of the FSC

#### NF modulation of the FSC in healthy volunteers

More than half of the reviewed studies engaged healthy volunteers to probe the feasibility of NF training in FSC regions. These studies sought to modify behavioral aspects such as emotion regulation (Sarkheil et al., 2015), motor performance (Hui et al., 2014; Blefari et al., 2015; Scharnowski et al., 2015; Al-Wasity et al., 2021), motivation (Li et al., 2018), working memory (Zhang et al., 2013; Sherwood et al., 2016), speech processing (Rota et al., 2009) and social avoidance (Lisk et al., 2020) with their results underscoring the relevance of the functional organization of the FSC. The studies that aimed to improve motor performance focused on regions in the motor subsystem, such as the M1, the SMA, and the PMA. Improvement of working memory was investigated by alteration in the DLPFC. The ACC as a part of the pain processing network (Qu et al., 2011) was targeted to improve pain perception, while the nucleus accumbens, an area underlying motivation and reward (Berridge, 2007), was the target region for motivation improvement.

NF studies involving healthy populations are crucially important given that the notion of mental health is not just the absence of mental illness. According to the World Health Organization (WHO), mental health is “a state of wellbeing in which the individual realizes his or her own abilities, can cope with the normal stresses of life, can work productively and fruitfully, and is able to make a contribution to his or her community” (World Health Organization, 2004). The main domains of mental health, including regulation of negative and positive valence systems, cognitive functioning, and social processes and interaction (Insel et al., 2010; Cuthbert and Insel, 2013), rely on the FSC (Dalley et al., 2008; Morris et al., 2016; Vaghi et al., 2017) and can be addressed by NF training.

#### NF modulation of the FSC in clinical populations

As shown in Figure 3, various clinical populations have been addressed in the reviewed NF studies. According to our review, patients with depression were successful in modulating self-esteem, brooding rumination, and depressive symptoms in general (Zahn et al., 2019; Takamura et al., 2020; Jaeckle et al., 2021; Keller et al., 2021; Taylor et al., 2022). Additionally, studies of patients suffering from anxiety reported clinical improvements in self-report of the anxiety level or control over contamination anxiety (Scheinost et al., 2013; Zilverstand et al., 2015; Zhao et al., 2019; Morgenroth et al., 2020). While SUD patients were found to successfully change their brain activity in the regulation targets, in some studies they were unable to significantly reduce craving (Kim et al., 2015; Kirsch et al., 2016; Karch et al., 2019). On the other hand, patients with Tourette's syndrome showed reduced symptoms after rt-fMRI NF, but no changes in the ROI were reported (Sukhodolsky et al., 2020). Altogether, the majority of the reviewed studies presented not only successful regulations of brain activity within the FSC, but also improvements in clinical symptoms. Collectively, these results suggest that NF based on the FSC may be a promising target for NF interventions in neuropsychiatric disorders. Nonetheless, the potential of NF in clinical populations needs to be fully exploited by means of further adjustments. As will be discussed later, there is room for improvement with respect to several aspects of study design of some of these studies.

### General study aspects

#### Sample size, controlling, blinding

NF-based interventions of FSC have been probed for a potential effect on treatment of psychiatric disorders, which highlight the importance of replicability and reliability for this line of research. Statistical power should be considered as an important marker for reliability of the results (Nord et al., 2017). Along with the systematically underpowered studies in neuroscience (Button et al., 2013; Stanley et al., 2018), almost half of the studies included in this review had only small sample sizes. A priori sample size estimation was performed in only four studies (Papoutsi et al., 2020; Garrison et al., 2021; Jaeckle et al., 2021; Weiss et al., 2022) and two studies performed a *post-hoc* power analysis (Mayeli et al., 2020; Morgenroth et al., 2020). Additionally, the studies lacked clarity with respect to effect size estimations. A recent meta-analysis (Fernández-Alvarez et al., 2022) has calculated an effect size of (Hedges' g) 0.303 for the efficacy of biofeedback for depressive symptoms. In the absence of the sample size estimations both under- and overpowered studies may occur, which causes a heterogeneity in the statistical power. Despite the well-known importance of a priori power analyses, and the low statistical power of psychological studies (Cohen, 1962), the reviewed studies failed to systematically calculate the statistical power a priori.

Pre-registrations in international online databases such as clinicaltrials.gov can motivate a priori power analyses. As a practice, pre-registration should be more widely used as it can also guarantee transparency among collaborators and prevent accusations of p-hacking. The oldest pre-registered study of the current review is from 2016 (Subramanian et al., 2016). While the number of pre-registered studies is on the rise, only 14 of the 43 studies between 2016 and now have mentioned pre-registrations. Another important aspect of probing an intervention method is the choice of an appropriate control condition. It is common knowledge that, in a study without a control condition, non-specific effects, such as placebo effects, motivation, and exercise effects (Sorger et al., 2019) cannot be excluded. For example, when monitoring symptom changes over time, the results of a non-controlled study should be closely examined with respect to natural recovery. Currently, there is no consensus regarding a control condition, which largely depends on particular aspects of the NF training design that need to be controlled. The current review has revealed the application of a variety of control conditions.

#### Age of the participants

The average age of the participants varied across the studies. Most studies examined adults, which reflects the manifestation age of the symptoms. Two studies looked at adolescents, one with patients with Tourette's syndrome and one with healthy volunteers. Critically, there is a strong association between dysfunction of frontostriatal regions and brain disorders in in adolescents, like SUD (Alegria et al., 2016; Bjork, 2020; Tervo-Clemmens et al., 2020), eating disorders (Marsh et al., 2009; Berner and Marsh, 2014) and anxiety disorders (Newman et al., 2016; Merz et al., 2018). In general, the age factor should be considered more carefully in selection of the study cohorts. Based on the developmental aspects of the FSC, there is a clear need for NF studies that focus on children or adolescents.

#### Feedback

Most of the reviewed studies used continuous feedback as opposed to intermittent feedback. However, the feedback time scale is a matter of discussion (Emmert et al., 2017). Comprehensiveness and timing account for the efficiency of the feedback-based training. Both rt-fMRI measurement techniques and implementations of psychological strategies impose limitations that are relevant for feedback timing. A timely and frequent feedback based on every MRI scan (continuous feedback) may facilitate learning. On the other hand, an intermittent feedback based on averaged MRI signals over an interval may provide more reliable and comprehensive information with respect to training. Empirical tests may help us find the best balance between comprehensiveness and frequency of feedback for each paradigm. We recommend integrating the identified moderators of the feedback-performance relationship (Kluger and DeNisi, 1996) in designing the feedback type in NF paradigms. For example, previous research has indicated an increased efficacy of positive feedback (Arbel et al., 2014) or feedback after successful trials (Chiviacowsky and Wulf, 2007), and normative feedback (Hartwell and Campion, 2016), which refers to information on one's performance compared to others. Interestingly, feedback has proved to be more effective when provided by a computer (Kluger and DeNisi, 1996).

#### Interval of training

There is no clear definition about NF training dose. However, repeating sessions have been suggested for different NF modalities (Fede et al., 2020; Domingos et al., 2021). Indeed, NF studies indicate that there is a correlation between the number of NF training sessions and the overall clinical effects in the treatment group (Trambaiolli et al., 2021).

In addition to the optimal number of sessions, the length of follow-up periods is important. Longer follow-up periods are desirable. According to clinical studies, effects following NF interventions last up to several months after the last NF session. Some studies have found effects to even improve over time (Mehler et al., 2018; Rance et al., 2018; Goldway et al., 2019).

### Future directions

Personalized interventions for people suffering from mental health conditions are currently the focus of research in this field. To this end, genomic-based and imaging-based subtyping are seen as the main avenues. NF can contribute to this effort by using individualized targeting in patients. This could lead to brain-based treatment by clarifying the neural basis of disordered behavior through real-time observations. Large NF studies using imaging-based subtyping may be a possibility for the future. Here, studies in healthy controls can be considered relevant for validation of controllability of specific targets. fMRI NF can also be used to clarify brain-behavior relationships that are critical to understanding and treating brain disorders. As a clinical neuroimaging tool, fMRI NF can potentially to be used for clinical diagnosis and to track the natural history of disease and the treatment progress. Finally, fMRI NF has the potential to make predictions not only about clinical outcomes but also about response to NF training. As this field is still lacking detailed investigation of neuronal mechanism of NF, augmentations by further imaging modalities such as positron emission tomography or single-photon spectroscopy might be a perspective for future. Importantly, fMRI NF can be integrated with other approaches aimed at modulating brain behavior pathways such as DBS and tDCS, offering another means of augmenting a desirable connectivity outcome.

The three-part organization of the FSC provides important reference for delineating brain circuits that can be differentially targeted for optimal intervention in various neuropsychiatric symptoms.

Another important question pertaining to future research has do with how the observed transient effects of NF may be translated to sustainable recovery in the clinical setting. The continuation of first-line treatments, such as psychotherapy, may be a useful strategy, as cognitive behavioral therapy is associated with changes in the frontostriatal connectivity (Yoshimura et al., 2017; Han et al., 2018). NF complements other existing neurotherapeutic technologies, including DBS and transcranial stimulation, by providing a non-invasive alternative for brain disorders. In addition, it may add value over psychotherapy alone by providing information about how and where cognitive changes in brain function are produced. Continuation of the previous treatment regime along with NF could be compared to single NF. Longitudinal follow-up studies with large samples are needed to probe the effect of psychotherapy and other strategies.

## Conclusion

Because of its importance in various psychiatric and neurological disorders, the FSC has been targeted by various neuromodulation techniques. NF, which describes the biofeedback of brain activity, can help individuals learn how to self-regulate their brain activity, thereby potentially inducing behavioral changes or improvements in clinical symptoms. The current review has shown that NF modulation of FSC structures has a great potential for interventions in neuropsychiatric disorders. The FSC can be divided into three spatially segregated loops, each being involved in different aspects of human behavior. We suggest that the topographical organization of the FSC should be considered in target selection for NF interventions. The network aspect of the FSC encourages investigation and selection of the functional connectivity as NF target. Further measures such as standardization of feedback, adjusting the training duration and interval and targeting the connectivity are expected to be helpful in optimizing the results.

## Data availability statement

The raw data supporting the conclusions of this article will be made available by the authors, without undue reservation.

## Author contributions

LO: conceptualization, study search, data selection/extraction, data interpretation, visualization, writing manuscript, and correction and revision. JM: data selection/extraction, data interpretation, and correction and revision of the manuscript. RG and IN: data interpretation and correction and revision of the manuscript. PS: conceptualization, data interpretation, correction and revision of the manuscript, and supervision. All authors contributed to the article and approved the submitted version.

## Funding

This work was funded by the Deutsche Forschungsgemeinschaft (DFG, German Research Foundation)–269953372/GRK2150 and 448334688.

## Conflict of interest

The authors declare that the research was conducted in the absence of any commercial or financial relationships that could be construed as a potential conflict of interest.

## Publisher's note

All claims expressed in this article are solely those of the authors and do not necessarily represent those of their affiliated organizations, or those of the publisher, the editors and the reviewers. Any product that may be evaluated in this article, or claim that may be made by its manufacturer, is not guaranteed or endorsed by the publisher.
